# Efficacy, Convenience, Safety and Durability of DTG-Based Antiretroviral Therapies: Evidence from a Prospective Study by the Italian MaSTER Cohort

**DOI:** 10.3390/v15040924

**Published:** 2023-04-06

**Authors:** Paolo Fusco, Paola Nasta, Eugenia Quiros-Roldan, Alice Tondinelli, Cecilia Costa, Chiara Fornabaio, Nicola Mazzini, Mattia Prosperi, Carlo Torti, Giampiero Carosi

**Affiliations:** 1 Infectious Diseases Unit, Department of Medical and Surgical Sciences, “Magna Graecia” University, 88100 Catanzaro, Italy; torti@unicz.it; 2 University Division of Infectious and Tropical Diseases, University of Brescia and Brescia ASST Spedali Civili Hospital, 25123 Brescia, Italy; p.nasta@infettivibrescia.it (P.N.); eugeniaquiros@yahoo.it (E.Q.-R.); 3 Fondazione Policlinico Universitario A. Gemelli IRCCS, 00168 Rome, Italy; alitondi@hotmail.com; 4 Infectious Diseases Unit, S. Maria Annunziata Hospital, 50012 Florence, Italy; cecilia.costa@uslcentro.toscana.it; 5 Infectious Diseases Unit, Cremona ASST Hospital, 26100 Cremona, Italy; chiara.fornabaio@asst-cremona.it; 6 M.I.S.I. Foundation, 25128 Brescia, Italy; mazzini.nicola@yahoo.com (N.M.); carosi@bsnet.it (G.C.); 7 Department of Epidemiology, College of Public Health and Health Professions and College of Medicine, University of Florida, Gainesville, FL 32603, USA; m.prosperi@ufl.edu

**Keywords:** HIV, dolutegravir, antiretroviral therapy, survival analysis, MaSTER cohort

## Abstract

*Background*: Dolutegravir (DTG) is recommended by international guidelines as a main component of an optimal initial regimen of cART (combination antiretroviral treatment) in people living with HIV (PLWH) and in case of switching for failure or optimization strategies. However, studies on the performance of DTG-containing regimens and indications for switching therapies in the long term are sparse. The purpose of this study was to evaluate prospectively the performance of DTG-based regimens, using the metrics of “efficacy”, “safety”, “convenience” and ‘’durability’’, among a nationally representative cohort of PLWH in Italy. *Methods*: We selected all PLWH in four centers of the MaSTER cohort who initiated a DTG-based regimen either when naïve or following a regimen switch between 11 July 2018 and 2 July 2021. Participants were followed until the outcomes were recorded or until the end of the study on 4 August 2022, whichever occurred first. Interruption was reported even when a participant switched to another DTG-containing regimen. Survival regression models were fitted to evaluate associations between therapy performance and age, sex, nationality, risk of HIV transmission, HIV RNA suppression status, CD4+ T-cell count, year of HIV diagnosis, cART status (naïve or experienced), cART backbone and viral hepatitis coinfection. *Results*: There were 371 participants in our cohort who initiated a DTG-based cART regimen in the time frame of the study. The population was predominantly male (75.2%), of Italian nationality (83.3%), with a history of cART use (80.9%), and the majority initiated a DTG-based regimen following a switch strategy in 2019 (80.1%). Median age was 53 years (interquartile range (IQR): 45–58). Prior cART regimen was based mostly on a combination of NRTI drugs plus a PI-boosted drug (34.2%), followed by a combination of NRTIs plus an NNRTI (23.5%). Concerning the NRTI backbone, the majority comprised 3TC plus ABC (34.5%), followed by 3TC alone (28.6%). The most reported transmission risk factor was heterosexual intercourse (44.2%). Total interruptions of the first DTG-based regimen were registered in 58 (15.6%) participants. The most frequent reason for interruption was due to cART simplification strategies, which accounted for 52%. Only 1 death was reported during the study period. The median time of total follow-up was 556 days (IQR: 316.5–722.5). Risk factors for poor performance of DTG-containing-regimens were found to be: a backbone regimen containing tenofovir, being cART naïve, having detectable HIV RNA at baseline, FIB-4 score above 3.25 and having a cancer diagnosis. By contrast, protective factors were found to be: higher CD4+ T-cell counts and higher CD4/CD8 ratio at baseline. *Conclusion:* DTG-based regimens were used mainly as a switching therapy in our cohort of PLWH who had undetectable HIV RNA and a good immune status. In this type of population, the durability of DTG-based regimens was maintained in 84.4% of participants with a modest incidence of interruptions mostly due to cART simplification strategies. The results of this prospective real-life study confirm the apparent low risk of changing DTG-containing regimens due to virological failure. They may also help physicians to identify people with increased risk of interruption for different reasons, suggesting targeted medical interventions.

## 1. Introduction

For people living with HIV (PLWH) with detectable viremia (HIV RNA), international guidelines recommend a combination antiretroviral therapy (cART) consisting of two nucleoside reverse transcriptase inhibitors (NRTIs) and one integrase strand transferase inhibitor (INSTI) as an optimal initial regimen unless otherwise indicated [1,2,3]. An INSTI-based regimen is also recommended in case of switching strategies for those with an undetectable HIV RNA [3]. Certain regimens may be contraindicated for some people who suffer from clinical conditions such as cardiovascular, kidney or liver diseases, opportunistic infections, or who are pregnant or planning a pregnancy, among other reasons [1,2]. Dolutegravir (DTG), has demonstrated good efficacy and tolerability in clinical trials [4] and in real life [5,6]; however, studies on performance of DTG-containing regimens and indications for switching therapies in the long-term are sparse. Recently, a retrospective study conducted in the Management Standardizzato di TErapia antiRetrovirale (MaSTER) cohort [7], showed that INSTI-based regimens were interrupted mostly due to safety concerns and suggested that DTG performed better in terms of durability compared to raltegravir (RAL) and elvitegravir (EVG). However, the results of this work were limited due to its retrospective nature and the shorter length of follow-up for DTG-containing regimens compared to RAL and EVG.

For this reason, a prospective study was designed, aimed at better evaluating in a multidimensional manner the performance of DTG-based regimens over a longer follow-up, controlling for potential confounders.

## 2. Materials and Methods

This is a prospective study nested in a multicenter, hospital-based cohort established in the mid-1990s that has currently enrolled over 24,000 people with HIV infection, i.e., the MaSTER cohort [8]. Data was collected from the enrolled persons’ medical, prescription, and laboratory records at regular time intervals (baseline, six months, and twelve months).

### 2.1. Inclusion Criteria

PLWH from four Italian hospital centers of the MaSTER cohort (Fondazione Policlinico Universitario A. Gemelli IRCCS, Rome, accounting for 19.1% of the total cohort; Brescia ASST Spedali Civili Hospital, Brescia, accounting for 57.1% of the total cohort; Cremona ASST Hospital, Cremona, accounting for 22.4% of the total cohort; S. Maria Annunziata Hospital, Florence, accounting for 1.4% of the total cohort) who initiated a DTG-based regimen, either when cART naïve or following a regimen switch, were included in the study between 11 July 2018 and 2 July 2021.

### 2.2. Outcomes

Participants were followed until the study outcomes were recorded or until the end of the study on 4 August 2022, whichever occurred first. Interruption was also reported when a participant switched to another DTG-containing regimen. Performance of DTG-based regimens was evaluated using the metrics of “efficacy”, “safety”, “convenience” and ‘’durability’’. An event due to efficacy concerns was defined as virological failure (HIV RNA > 50 copies/mL at least six months after initiating therapy), whether or not therapy interruption occurred. Events due to safety concerns were defined as a newly occurring laboratory alteration of grades 3–4 and/or clinical progression whether or not there was a therapy interruption. Grade 3+ laboratory alterations were defined using the following measurements: aspartate transaminase (AST) > 260 units/L, alanine transaminase (ALT) > 235 units/L, bilirubin > 2.6 mg/dL, cholesterol ≥ 275 mg/dL, triglycerides > 750 mg/dL, glycemia > 250 mmol/L, creatinine > 1.9 mg/dL, calcium < 7 mg/dL, and iron < 2 g/dL. Conditions considered as clinical progression of HIV disease included a diagnosis of acquired immunodeficiency syndrome (AIDS), cancer, hepatic cirrhosis defined as a FIB-4 ≥ 3.25 [9], an ischemic cardiovascular event, kidney disease defined as eGFR (estimated glomerular filtration rate) < 89 mL/min, and any-cause death. An event due to convenience concerns was defined as having HIV RNA > 50 copies/mL and a laboratory alteration (composite outcome of efficacy and safety), whether or not there was a therapy interruption. Durability was defined as the total course of treatment until interruption regardless of tolerability, HIV RNA level, laboratory alteration, or clinical progression.

### 2.3. Statistical Approach

Kaplan–Meier estimators, univariate and multivariable Cox proportional hazards regression models were fitted to associate incidence of efficacy, convenience, safety and durability (interruption) events of DTG therapies with demographics (age, sex, and nationality), risk factors for HIV transmission (heterosexual intercourse, people who inject drugs (PWID), men who have sex with men (MSM), or other), cART status (naïve or experienced), CD4+ T-cell count, CD8 + T-cell count, CD4/CD8 ratio, HIV RNA at baseline, presence of hepatitis B infection or hepatitis C antibodies (HCVAb), cART backbone, eGFR, FIB-4 score [9], Framingham score [10], prior diagnosis of cancer, and year of starting DTG. Missing values in the multivariable analysis were imputed using random forests, while case deletion was used in univariate analysis. In the multivariable models, each exposure of interest above defined was adjusted by a set of covariates identified through the generalized adjustment criterion on a directed acyclic graph agreed by the co-authors (Figure S1).

All analyses and data visualizations were performed in R statistical programming software using the following packages: survival [11], dagitty [12], missforest [13].

## 3. Results

### 3.1. Patient Characteristics

There were 371 participants who initiated a DTG-based cART regimen in four centers of the MaSTER cohort during the time frame of the study. Median age was 53 years (IQR: 45–58). The majority of the study population were male (75.2%), of Italian nationality (83.3%), with a history of cART use (80.9%) and the majority of them initiated a DTG-based regimen after switching from previous regimens in 2019 (80.1%) (Table 1).

Among experienced people, prior cART regimens were mostly based on a combination of nucleoside/nucleotide reverse transcriptase inhibitors (NRTIs) in addition to a boosted protease inhibitor (PI) (34.2%) followed by a combination of NRTIs plus a non nucleoside reverse transcriptase inhibitor (NNRTI) (23.5%) (Table 1). Other prior cART regimens (18.3%) included a miscellaneous combination of drugs of which previous INSTI exposure accounted for 8.1% (Table S1). The most frequent NRTI backbone was represented by lamivudine (3TC) plus abacavir (ABC) (34.5%), followed by 3TC alone (28.6%), and FTC in addition to tenofovir disoproxil fumarate (TDF) (12.9%) (Table 1). Dual therapies were more frequent among experienced participants compared to naïve people (43.7% vs. 15.5%), while tenofovir containing backbones were more used by naïve participants compared to experienced people (59.2% vs. 12.7%) (Tables S2 and S3).

The reported risk factors for HIV transmission were: heterosexual intercourse (44.2%), MSM intercourse (27.5%), and intravenous drug use (21.6%) (Table 1).

HBsAg chronic carriers and/or positive HCV Ab participants accounted for 4.3% of the population. A previous diagnosis of cancer was present in 33 participants (8.9%) (Table 1).

Median HIV RNA at baseline was 1.7 Log_10_ copies/mL (IQR: 1.7–2.09). Median CD4+ T-cell count at baseline was 640 cells/mm^3^ (IQR: 420–856), CD8 + T-cell count at baseline was 806 cells/mm^3^ (IQR: 572–1112), and CD4/CD8 ratio at baseline was 0.76 (IQR: 0.48–1.1) (Table 1). Naïve participants had higher baseline HIV RNA compared to experienced people (5.05 Log_10_ copies/mL (IQR: 4.4–5.38) vs. 1.7 Log_10_ copies/mL (IQR: 1.7–1.7)) and lower values of CD4+ T-cell count (207 cells/mm^3^ (IQR: 54–463) vs. 700 cells/mm^3^ (IQR: 536–897)) and CD4/CD8 ratio (0.18 (IQR: 0.09–0.52) vs. 0.83 (IQR: 0.59–1.18)) at baseline (Tables S2 and S3).

At baseline, median FIB-4 score was 1.08 (IQR: 0.74–1.55), median eGFR was 92.34 mL/min (IQR: 79.33–104.02), and median Framingham score was 12 (IQR: 10–15) (Table 1).

### 3.2. DTG Regimen Performance and Rates of Interruption

Regarding DTG performance in the study cohort, 81 efficacy events (25.2%), 107 convenience events (33.2%), 76 safety events (23.6%) and 58 durability events (18.0%) occurred. These events led to a DTG-containing regimen interruption or change (durability events) in 58 (15.6%) people overall, over a median follow-up of 556 days (IQR: 316.5–722.5). The most frequent reason for interruption was cART simplification, which accounted for 52%, followed by a 29.3% rate of interruption due to toxicity (mostly rash, hypersensitivity reactions and nephrotoxic effects) and a 6.9% interruption due to virological failure. Participants lost to follow-up were only 2 (3.5%), and only 1 patient died during the study period (Table 1).

**Figure 1:**
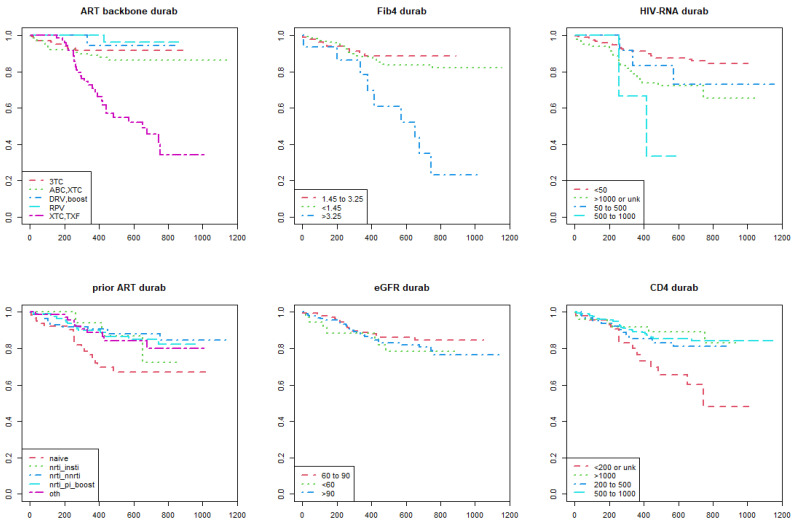
Kaplan–Meier survival curves show the time to a durability event (interruption) of dolutegravir-based antiretroviral therapies among people with HIV enrolled in the Italian MaSTER cohort. Plots are stratified by: ART backbone, FIB4 score, HIV RNA, previous ART, eGFR, CD4+ T-cell count. DURAB, durability; ART, antiretroviral treatment; NRTI, nucleos(t)ide reverse transcriptase inhibitors; NNRTI, non-nucleos(t)ide reverse transcriptase inhibitors; INSTI, integrase strand inhibitors; PI, protease inhibitors; RPV, rilpivirine; TXF, tenofovir alafenamide or tenofovir disoproxil fumarate; XTC, emtricitabine or lamivudine; 3TC, lamivudine; ABC, abacavir; DRV, darunavir; Boost, ritonavir-boosted regimens; OTH, other; eGFR, estimated Glomerular Filtration Rate. *X*-axis: time (days); *Y*-axis: survival rate (%).

**Figure 2:**
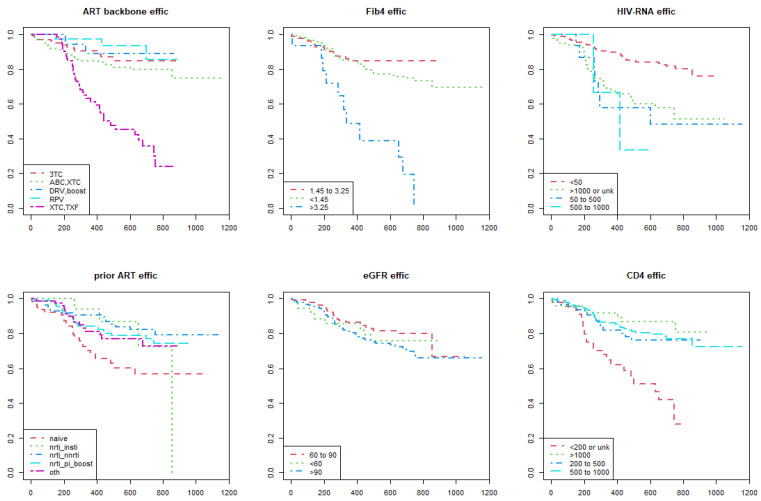
Kaplan–Meier survival curves show the time to an efficacy event of dolutegravir-based antiretroviral therapies among persons with HIV enrolled in the Italian MaSTER cohort. Plots are stratified by: ART backbone, FIB4 score, HIV RNA, previous ART, eGFR, CD4+ T-cell count. EFFIC, efficacy; ART, antiretroviral treatment; NRTI, nucleos(t)ide reverse transcriptase inhibitors; NNRTI, non-nucleos(t)ide reverse transcriptase inhibitors; INSTI, integrase strand inhibitors; PI, protease inhibitors; RPV, rilpivirine; TXF, tenofovir alafenamide or tenofovir disoproxil fumarate; XTC, emtricitabine or lamivudine; 3TC, lamivudine; ABC, abacavir; DRV, darunavir; Boost, ritonavir-boosted regimens; OTH, other; eGFR, estimated Glomerular Filtration Rate. *X*-axis: time (days); *Y*-axis: survival rate (%).

**Figure 3:**
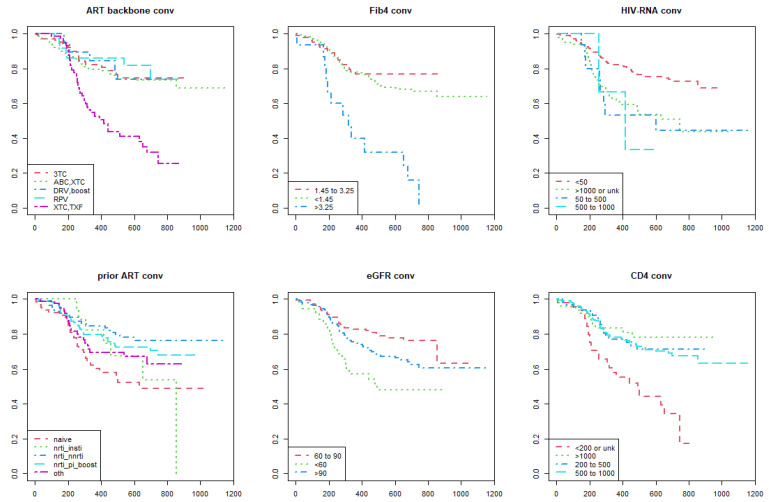
Kaplan–Meier survival curves show the time to a convenience event of dolutegravir-based antiretroviral therapies among people with HIV enrolled in the Italian MaSTER cohort. Plots are stratified by: ART backbone, FIB4 score, HIV RNA, previous ART, eGFR, CD4+ T-cell count. CONV, convenience; ART, antiretroviral treatment; NRTI, nucleos(t)ide reverse transcriptase inhibitors; NNRTI, non-nucleos(t)ide reverse transcriptase inhibitors; INSTI, integrase strand inhibitors; PI, protease inhibitors; RPV, rilpivirine; TXF, tenofovir alafenamide or tenofovir disoproxil fumarate; XTC, emtricitabine or lamivudine; 3TC, lamivudine; ABC, abacavir; DRV, darunavir; Boost, ritonavir-boosted regimens; OTH, other; eGFR, estimated Glomerular Filtration Rate. *X*-axis: time (days); *Y*-axis: survival rate (%).

**Figure 4:**
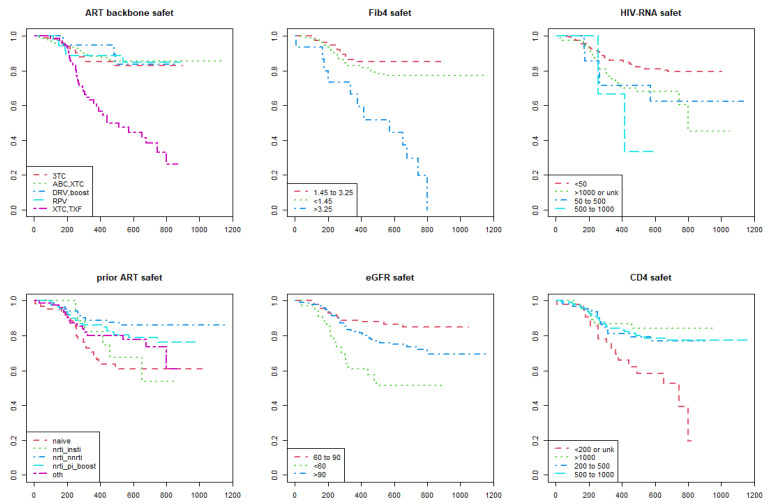
Kaplan–Meier survival curves show the time to a safety event of dolutegravir-based antiretroviral therapies among persons with HIV enrolled in the Italian MaSTER cohort. Plots are stratified by: ART backbone, FIB4 score, HIV RNA, previous ART, eGFR, CD4+ T-cell count. SAF, safety; ART, antiretroviral treatment; NRTI, nucleos(t)ide reverse transcriptase inhibitors; NNRTI, non-nucleos(t)ide reverse transcriptase inhibitors; INSTI, integrase strand inhibitors; PI, protease inhibitors; RPV, rilpivirine; TXF, tenofovir alafenamide or tenofovir disoproxil fumarate; XTC, emtricitabine or lamivudine; 3TC, lamivudine; ABC, abacavir; DRV, darunavir; Boost, ritonavir-boosted regimens; OTH, other; eGFR, estimated Glomerular Filtration Rate. *X*-axis: time (days); *Y*-axis: survival rate (%).

As shown in the Kaplan–Meier estimate curves, lower performance of DTG-based regimens according to the metric of durability (Figure 1) and efficacy (Figure 2) were found in the following categories of participants: (i) those who were prescribed a NRTI backbone containing tenofovir; (ii) FIB4 score > 3.25; (iii) higher HIV RNA levels; (iv) CD4+ T-cell count less than 200 cells/mm^3^; (v) previously cART naïve. In addition to the categories listed above, the Kaplan–Meier survival analysis showed lower performance of DTG-based regimens according to the metric of convenience (Figure 3) and safety (Figure 4) in the following participants: (i) those switched to a DTG-containing regimen from a previous cART containing another INSTI; (ii) those with eGFR below 60 mL/min.

### 3.3. Associations with Efficacy Events

With univariate analysis, greater hazards of efficacy event occurrence were observed among individuals with a backbone regimen containing emtricitabine plus tenofovir, naïve people at baseline, those with a detectable HIV RNA and those with FIB-4 above 3.25. Participants switched to a DTG-containing regimen from a previous cART not containing an INSTI had a lower hazard of efficacy event occurrence, as did people with higher CD4+ T-cell count and higher CD4/CD8 ratio at baseline (Table 2).

Using the multivariable model, significantly higher hazards of efficacy event occurrence were found in participants who were prescribed emtricitabine plus tenofovir and with detectable HIV RNA at baseline (Table 3); experienced people switched from a cART regimen had a lower hazard of efficacy event occurrence compared to naïve participants (Table 3).

### 3.4. Associations with Convenience Events

With univariate analysis, higher hazards of a convenience event occurring were observed among individuals with a backbone regimen containing emtricitabine plus tenofovir, those who were naïve, those with a higher viral load at baseline, FIB-4 at baseline above 3.25, positive HBsAg, and eGFR at baseline below 60 mL/min (Table 2). Lower hazards of a convenience event occurring were observed among experienced people and those having higher CD4+ T-cell count and higher CD4/CD8 ratio at baseline.

Using the multivariable model, significantly higher hazards of a convenience event occurring were indicated among individuals with a backbone regimen containing emtricitabine plus tenofovir and higher HIV RNA at baseline. Experienced people had lower hazards of occurrence of an event due to convenience (Table 3).

### 3.5. Associations with Safety Events

With univariate analysis, higher hazards of occurrence of events due to safety concerns were observed among individuals with a backbone regimen containing emtricitabine plus tenofovir, among participants with a higher viral load at baseline, FIB-4 above 3.25 and eGFR at baseline below 60 mL/min. Experienced people, having higher CD4+ T-cell count and higher CD4/CD8 ratio at baseline and those having an increased Framingham score at baseline had lower hazards of occurrence of events due to safety (Table 2).

Using the multivariable model, significantly higher hazards of occurrence of a safety event were maintained only among individuals with a backbone regimen containing emtricitabine plus tenofovir, while lower hazards were maintained in experienced people (Table 3).

### 3.6. Associations with Durability Events

With univariate analysis, hazards of interruption or therapy change (durability) were significantly greater in the following participants: those receiving a backbone regimen containing emtricitabine plus tenofovir, cART naïve participants, those with higher HIV RNA, FIB4 at baseline above 3.25, and cancer. Lower hazards of interruption were observed among people with higher CD4+ T-cell count, higher CD4/CD8 ratio at baseline, switching from cART not containing an INSTI compared to naïve people and having an increased Framingham score at baseline (Table 2).

Using the multivariable model, significant higher hazards of interruption were maintained among individuals prescribed a backbone regimen containing emtricitabine plus tenofovir and with higher HIV RNA at baseline. Experienced participants prescribed cART regimens not containing an INSTI had lower hazards of interruption compared with cART naïve people for the metric of durability (Table 3).

**Figure 5:**
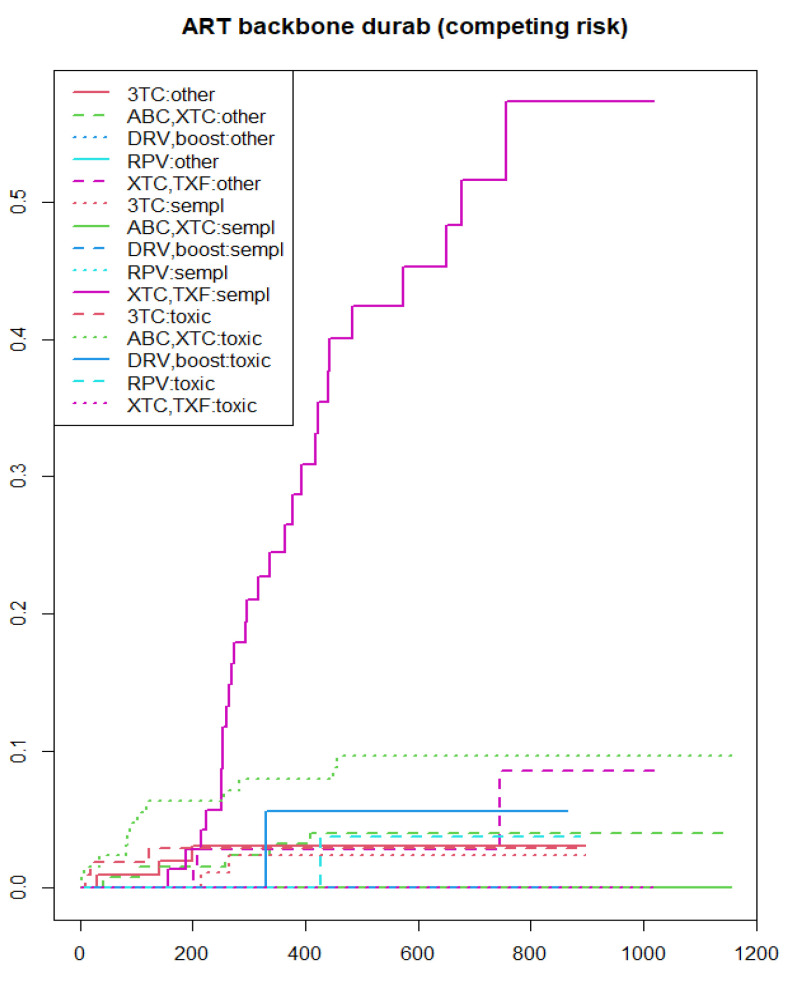
Kaplan–Meier survival curve, obtained by a competing risk analysis, shows how the tenofovir-containing backbone was mainly interrupted due to simplification strategies. DURAB, durability; SEMPL, simplification; ART, antiretroviral treatment; TOXIC, toxicity; RPV, rilpivirine; TXF, tenofovir alafenamide or tenofovir disoproxil fumarate; XTC, emtricitabine or lamivudine; 3TC, lamivudine; ABC, abacavir; DRV, darunavir; Boost, ritonavir-boosted regimens. *X*-axis: time (days); *Y*-axis: survival rate (%).

The backbone regimen containing emtricitabine plus tenofovir was mainly interrupted due to simplification strategies as we observed in a competing risk analysis (Figure 5).

## 4. Discussion

In this study, we prospectively evaluated the performance of DTG-based regimens in a multi-dimensional way, using the metrics of “efficacy”, “convenience”, “safety” and ‘’durability’’, in a nested study of the Italian MaSTER cohort. The population examined had a median age of 53 years and was mainly composed by PLWH experienced for cART, since naïve participants were only 19% of the overall population. The majority of participants had undetectable HIV RNA and a high CD4+ T-cell count (median of 640 cells/mm^3^), reflecting that DTG-containing regimens were mainly prescribed (particularly in the year 2019) in order to optimize cART in those already responding to treatment.

The results of the study in terms of independent predictors of the outcomes for each of the study metrics are summarized in Table 4.

Concerning the metric of efficacy, our results suggest that potential risk factors for the occurrence of an efficacy event were: **(a) the backbone regimen containing emtricitabine plus tenofovir.** Regarding this finding, however, we did not directly compare the efficacy of the backbone regimens with each other and we are not able to fully explain our results. They most likely reflect the extensive use of tenofovir in cART combinations, expecially in naïve patients, therefore increasing the possibility of its statistical association with detectable viraemia; **(b) the condition of being cART naïve**, probably because of a “selection of the fittest” effect, because treatment experienced participants (mostly with undetectable HIV RNA) were those on stable treatment and therefore with consolidated adherence to cART. In addition, naïve participants with higher baseline HIV RNA (Tables S2 and S3) may need more time on cART to obtain undetectable HIV RNA. Thus, the condition of being naïve was associated with a higher probability of having detectable viremia after six months of follow-up compared to experienced people who already responded to treatment and in whom cART was previously optimized; **(c) having higher HIV RNA at baseline**, as already found in a previous work of the MaSTER cohort [7], pointing out the importance of carefully monitoring these people for HIV RNA, even those using DTG-containing regimens, who may run the risk of low level viremia after six months of treatment [14]; **(d) FIB4 score > 3.25**. Regarding this finding, the correlation between poor cART adherence and increased values of FIB4 score is known to grow in the eventuality of there being detectable viremia in people with liver fibrosis [15]. These findings provide further support to improve medical interventions and counseling with the aim of improving cART adherence in this population, minimizing the risk of virological failure. In contrast, protective factors for the occurrence of an efficacy event were: **(a) previous cART not containing an INSTI**. This finding seems to confirm the appropriateness of switching to an INSTI-based regimen to simplify regimens containing alternative drugs without compromising the virological control in line with the conclusions by Raffi F. et al. [6], who reported good efficacy outcomes after switching to INSTI regimens in virologically suppressed PLWH who were taking PI/r or NNRTI containing-regimens; **(b) higher CD4+ T-cell count and higher CD4/CD8 ratio at baseline**, in line with the findings of a study nested in a cohort of South African PLWH in which lower baseline CD4+ T-cell count was associated with a greater propensity toward virological failure, underlining the importance of preserving the immune system even in people prescribed well tolerated and effective drugs [16].

Concerning the metric of safety, we found as potential risk factors: **(a) the backbone regimen containing emtricitabine plus tenofovir,** suggesting the importance of taking into consideration the potential toxicity of NRTIs in composing the backbone of the cART regimens. For example, the risk of renal and bone toxicity associated with the use of TDF is well known [17,18]; **(b) the condition of being cART naïve**, probably, for the same reasons already mentioned for the metric of efficacy; **(c) having higher HIV RNA at baseline**, confirming the importance of maintaining HIV RNA control for prevention of the clinical events, even independently from the immunological status of the patient. For example, a previous study performed in the MaSTER cohort demonstrated how a longer delay from HIV diagnosis to HAART was an independent predictor of new AIDS-defining events and deaths. Thus, a longer time spent with lower HIV RNA plasma levels was found to be protective from clinical progression [19]; **(d) eGFR at baseline below 60 mL/min and FIB4 score > 3.25**, reflecting the importance of care for PLWH with kidney failure and advanced liver disease, ensuring regular checkups and medical interventions to avoid an unfavorable outcome. In contrast, protective factors for occurrence of safety events were: **(a) previous cART not containing an INSTI**, in line with what already discussed for the metric of efficacy and the findings of a review by Raffi F. et al., in which switching to INSTI regimens, in virologically suppressed PLWH, was associated with improved tolerability and greater reported patient satisfaction and outcomes compared to other ARV drug classes [6]; **(b) higher CD4+ T-cell count and higher CD4/CD8 ratio at baseline**, according to previous study showing the importance of having higher basal CD4+ T-cell count and a good CD4/CD8 ratio to avoid AIDS-defining events and deaths [19,20]; **(c) an increased Framingham score at baseline**, probably supporting the overall safety profile of using INSTI in PLWH with high risk of cardiovascular adverse events. A decreased risk of cardiovascular disease with the use of INSTI containing regimens compared to other ARV drug classes is described in a retrospective study of a large cohort of PLWH [21]. By contrast, the findings from the RESPOND cohort consortium showed how INSTI initiation was associated with an early onset and excess incidence of cardiovascular disease in the first 2 years of exposure [22]. These preliminary findings require analysis in larger randomized controlled trials to clarify the relationship between INSTI use and cardiovascular adverse events.

Since the metric of convenience constitutes a composite outcome of efficacy and safety, in general it shares the same risk and protective factors. However, **the status of chronic HBsAg carrier** at baseline was also found to be a risk factor for occurrence of a convenience event. It is well known that people with HIV and HBV co-infection with controlled plasma viral load still have an increased risk of liver disease progression, liver-related mortality, and overall mortality compared to people with either HIV or HBV alone [23,24,25]. Moreover, a recent study demonstrated liver as an HIV reservoir in PLWH on cART, showing persistence of HIV DNA in hepatocytes of PLWH on antiretroviral therapy, resulting in a status of chronic inflammation that could lead to adverse liver outcomes [26]. In addition to this, a positive association between liver fibrosis and suboptimal cART was observed in previous studies [15]. Thus, close attention should be paid to co-infected patients in order to optimize therapy and implement appropriate medical interventions.

In the present cohort, durability appeared to work in favor of DTG based regimens with 58/371 (15.6%) participants stopping the initial regimen, especially if one considers that changing any drugs in the regimen was considered as an outcome. Moreover, durability events were mostly due to cART simplification which explained half of the interruptions, followed by treatment modification or interruption due to drug regimen toxicity, occurring in 29.3% of interruptions; this is in line with the results of previous studies [27] and suggests the effectiveness of a proactive treatment switch in the modern antiretroviral treatment era. Importantly, interruptions due to virological failure were few, accounting for only 1.1% (4/371) of total interruptions, confirming low rates of virological failure of DTG-based regimens.

When exploring potential risk factors associated with the metric of durability, we found that the following factors were associated with an increased risk of treatment interruption: **(a) the backbone regimen containing tenofovir**, mainly due to simplification strategies as shown in the KM estimates resulting from competitive risk analysis (Figure 5). We can hypothesize that this reflects the choice of removing tenofovir disoproxil fumarate to improve renal and bone toxicities [17,18]. An alternative explanation could be related to treatment optimization by changing treatment to tenofovir alafenamide in those already on tenofovir disoproxil fumarate to prevent renal and bone deterioration, given the comparative renal safety advantage of TAF versus TDF in PLWH [28]; **(b) the condition of being cART naïve**, in apparent accordance to the findings of a multicenter Italian cohort in which treatment in naïve people showed a lower probability of maintaining DTG containing regimens at three and five years compared to treatment-experienced PLWH [27]. Since, in our cohort, DTG containing regimens were mostly interrupted due to simplification strategies, we can suppose that naïve participants (as well as experienced ones) interrupted their regimen for simplification reasons, either to reduce pill burden or because of drug related toxicity or tolerability reasons; **(c) having higher HIV RNA at baseline,** confirming and supporting the findings already discussed about the metrics of efficacy, safety and convenience; **(d) a FIB-4 at baseline above 3.25 compared to those whose FIB-4 was in the range from 1.45 to 3.25**, confirming results of previous studies in which a positive association between liver fibrosis and suboptimal cART was observed [15] and encouraging physicians to start earlier cART [29,30]; **(e) a baseline cancer diagnosis** was associated with higher hazards of interruptions of DTG-containing therapies. It may be that antiretroviral therapy was optimized to minimize drug interactions and chemotherapy-related toxicities. It is known that PLWH are at increased risk of cancer when compared to the general population, particularly for malignancies driven by viral and bacterial co-infections, although they also have excess risk of infection-unrelated malignancies [31,32]. Recent randomized data (START Trial) indicated that immediate cART initiation reduces risk of cancer during early HIV infection before the development of overt immunosuppression [33]. Despite the increased toxicity and drug–drug interactions during cancer treatment, deferring cART during chemotherapy is unfavorable in PLWH with cancer and can lead to a poorer outcome; in general, any treatment interruption is not recommended during cancer treatment [34], with INSTI drugs appearing to be a good therapeutic choice in people with hematological malignances or those receiving various chemotherapeutic agents [35]. In contrast, protective factors for durability of DTG containing regimens were found to be: **(a) previous cART not containing an INSTI and previous cART containing other prior cART regimens** that were composed of single class, triple class, non-boosted PI regimens and combinations including both INSTI and PI in which previous INSTI exposure accounted for only 8.1% (Table S1); **(b) higher CD4+ T-cell count and CD4/CD8 ratio at baseline**, confirming results observed for the metrics of efficacy, convenience and safety, and reflecting the importance of earlier diagnosis and cART introduction to restore or maintain a better immune status; **(c) an increased Framingham score at baseline**, probably indicating a good durability of DTG based regimens in participants with high cardiovascular risk, as we have already discussed for the metric of safety.

Lastly, in this cohort, no statistically significant associations were observed between the outcomes analyzed and the demographic (age, gender, and nationality) or epidemiological (heterosexual, PWID, MSM, or other risk factors for HIV transmission) patient characteristics.

This study is affected by several limitations. Firstly, for some parameters analyzed, there was a large confidence interval, due to fragmented data in the follow-up period; this was probably due to the COVID-19 pandemic which occurred concomitantly with enrollment and follow-up of participants, reducing the precision of our estimates. Secondly, since the study is not randomized, it is affected by the intrinsic limitations in any observational datasets, including possible confounding by indication biases. Third, although INSTI drugs, particularly DTG, showed better efficacy, safety and durability compared to PI drugs in improving lipid profiles [5,36,37], we did not evaluate metabolic and cardiological toxicities linked to this drug class [22,38]. These adverse events need to be evaluated in the near future by larger studies, in order to implement educational and multidimensional interventions to prevent metabolic alterations in PLWH, especially for those with particular risk factors such as alcohol abuse, osteoporosis, previous AIDS events, and polypharmacy [39]. Further, we did not assess changes of specific drugs in the study regimens, types of drugs in the regimens to which the participants were switched, or the outcome of these regimens after the switch; more studies are needed to address these points. Notwithstanding the above limitations, we feel that the prospective design, the prolonged follow-up (median: 556 days), the “real-life” conditions, and the multi-dimensional evaluation with several outcomes may provide interesting observations on the way DTG was used and how the regimens containing this drug should be optimized.

## 5. Conclusions

This prospective “real-life” study showed that DTG-based regimens were started particularly in the year 2019 mainly as a switching therapy, and in experienced PLWH already responding to cART in terms of viral suppression. In this type of population, durability appeared to work in favor of DTG-based regimens that were maintained without any changes in 84.4% of participants, with only a low frequency of interruptions, mostly for reasons of cART simplification. Risk factors for interruption were: a backbone regimen containing tenofovir (driven mostly by simplification strategies), being cART naïve, a higher viral load at baseline, a high FIB4 score and having cancer. By contrast, greater CD4+ T-cell count and CD4/CD8 ratio at baseline appeared to be protective factors. Therefore, although uncontrolled, the present results confirm the apparent low risk of changing DTG-containing regimens due to virological failure and help physicians to identify people with an increased risk of interruption. Targeted medical interventions are important to further maximize performance of the regimens in terms of efficacy, convenience, safety and durability.

## Figures and Tables

**Table 1:** Characteristics of the full study population.

Characteristics of Full Population (N = 371)	N° or Median	% or (IQR)
**Age (years)**	53	(45–58)
**Female sex**	92	24.8%
**Country (Italy)**	309	83.3%
**Year of starting DTG**		
2018	21	5.7%
2019	297	80.1%
2020	42	11.3%
2021	11	3.0%
**Prior cART**		
Naïve	71	19.1%
NRTI/INSTI	18	4.9%
NRTI/NNRTI	87	23.5%
NRTI/PI-boosted	127	34.2%
Others	68	18.3%
**cART backbone**		
FTC/TDF	48	12.9%
3TC	106	28.6%
3TC/ABC	128	34.5%
RPV	36	9.7%
Cobi/DRV	18	4.9%
FTC/TAF	27	7.3%
3TC/TDF	5	1.3%
ABC/FTC	1	0.3%
DRV/RTV	2	0.5%
**Mode of transmission**		
Other	25	6.7%
MSM	102	27.5%
PWID	80	21.6%
Heterosexual	164	44.2%
**HBsAg chronic carriers**	7	1.9%
**Positive HCV Ab participants**	9	2.4%
**Prior/baseline cancer**	33	8.9%
**Baseline HIV RNA Log_10_ copies/mL**	1.7	(1.7–2.09)
**Baseline CD4 T-cells/mm^3^**	640.5	(420.25–856.25)
**Baseline CD8 T-cells/mm^3^**	806.5	(571.75–1112.5)
**Baseline CD4/CD8 ratio**	0.76	(0.48–1.1)
**Baseline FIB-4 score**	1.08	(0.74–1.55)
**Baseline eGFR (ml/min)**	92.34	(79.33–104.02)
**Baseline Framingham score**	12	(10–15)
**DTG regimens performance**		
Efficacy events	81	25.2%
Convenience events	107	33.2%
Safety events	76	23.6%
Durability events	58	18.0%
**Total participant interruptions**	58	15.6%
Due to cART simplification	30	51.7%
Due to toxicity	17	29.3%
Due to virological failure	4	6.9%
Due to loss to follow-up	2	3.5%
Due to other reasons	4	6.9%
Death	1	1.7%
**Total follow up time (days)**	556	(316.5–722.5)

IQR, interquartile range; DTG, dolutegravir; cART, combination antiretroviral treatment; NRTI, nucleos(t)ide reverse transcriptase inhibitors; NNRTI, non-nucleos(t)ide reverse transcriptase inhibitors; INSTI, integrase strand inhibitors; PI, protease inhibitors; FTC, emtricitabine; RPV, rilpivirine; TDF, tenofovir disoproxil fumarate; 3TC, lamivudine; ABC, abacavir; TAF, tenofovir alafenamide fumarate; DRV, darunavir; RTV, ritonavir; COBI, cobicistat; PWID, people who inject drugs; MSM, men who had sex with men; eGFR, estimated Glomerular Filtration Rate.

**Table 2:** Hazards of treatment interruption in relation to each of the outcome events (univariate analysis).

	Hazards of Interruption (95% Confidence Interval; *p*-Value) Due to:			
	Efficacy	Convenience	Safety	Durability
**Age (per 10 years old older)**	0.89 (0.72–1.10; 0.30)	0.99 (0.82–1.19; 0.91)	0.99 (0.80–1.24; 0.96)	0.98 (0.76–1.26; 0.88)
**Sex**				
Female	*Reference category*	*Reference category*	*Reference category*	*Reference category*
Male	0.95 (0.57–1.58; 0.85)	0.78 (0.51–1.18; 0.25)	0.81 (0.49–1.34; 0.41)	0.92 (0.51–1.65; 0.77)
**Nationality**				
Non-Italian	*Reference category*	*Reference category*	*Reference category*	*Reference category*
Italian	0.64 (0.38–1.08; 0.10)	0.65 (0.41–1.04; 0.07)	0.74 (0.42–1.30; 0.29)	0.71 (0.37–1.34; 0.29)
**Risk group**				
Heterosexual	*Reference category*	*Reference category*	*Reference category*	*Reference category*
PWID	1.05 (0.59–1.87; 0.87)	1.14 (0.70–1.84; 0.61)	1.35 (0.79–2.30; 0.28)	0.93 (0.48–1.81; 0.82)
MSM	1.35 (0.81–2.27; 0.25)	1.18 (0.74–1.87; 0.49)	0.87 (0.48–1.56; 0.63)	1.14 (0.62–2.07; 0.68)
Other	1.11 (0.34–2.85; 0.83)	1.20 (0.54–2.67; 0.66)	0.91 (0.32–2.57; 0.86)	0.27 (0.04–1.98; 0.20)
**Diagnosis year**	0.69 (0.41–1.16; 0.16)	0.83 (0.53–1.29; 0.40)	1.17 (0.71–1.92; 0.54)	1.00 (0.56–1.82; 0.98)
**cART backbone**				
3TC	*Reference category*	*Reference category*	*Reference category*	*Reference category*
ABC/XTC	1.30 (0.65–2.59; 0.46)	1.05 (0.60–1.83; 0.86)	0.74 (0.37–1.51; 0.41)	1.31 (0.57–3.05; 0.53)
DRV-boosted	0.62 (0.14–2.77; 0.53)	0.93 (0.35–2.48; 0.88)	0.77 (0.22–2.70; 0.69)	0.47 (0.06–3.77; 0.48)
RPV	0.55 (0.16–1.95; 0.35)	0.80 (0.34–1.90; 0.62)	0.80 (0.29–2.21; 0.66)	0.27 (0.03–2.19; 0.22)
XTC/TXF	**4.85 (2.53–9.28; <0.01)**	**3.10 (1.82–5.30; <0.01)**	**3.86 (2.08–7.14; <0.01)**	**5.71 (2.62–12.43; <0.01)**
**cART status**				
Experienced	*Reference category*	*Reference category*	*Reference category*	*Reference category*
Naïve	**2.26 (1.39–3.67; <0.01)**	**1.97 (1.28–3.04; <0.01)**	**2.08 (1.26–3.45; <0.01)**	**2.49 (1.42–4.36; <0.01)**
**Previous cART**				
Naïve	*Reference category*	*Reference category*	*Reference category*	*Reference category*
NRTI/INSTI	0.49 (0.17–1.40; 0.18)	1.72 (0.31–1.64; 0.43)	0.85 (0.34–2.12; 0.73)	0.49 (0.14–1.65; 0.25)
NRTI/NNRTI	**0.35 (0.18–0.67; <0.01)**	**0.37 (0.21–0.67; <0.01)**	**0.30 (0.14–0.62; <0.01)**	**0.35 (0.16–0.74; <0.01)**
NRTI/PI-boosted	**0.47 (0.26–0.83; <0.01)**	**0.50 (0.30–0.84; <0.01)**	**0.48 (0.26–0.88; 0.02)**	**0.41 (0.21–0.80; <0.01)**
Other	0.52 (0.27–1.00; 0.05)	0.65 (0.37–1.13; 0.13)	0.63 (0.33–1.21; 0.16)	**0.44 (0.20–0.96; 0.04)**
**Positive HBsAg at baseline**				
NO	*Reference category*	*Reference category*	*Reference category*	*Reference category*
YES	1.83 (0.58–5.80; 0.31)	**2.70 (1.10–6.64; 0.03)**	2.65 (0.97–7.25; 0.06)	1.50 (0.37–6.14; 0.57)
**Positive HCV Ab at baseline**				
NO	*Reference category*	*Reference category*	*Reference category*	*Reference category*
YES	0.87 (0.53–1.45; 0.60)	0.94 (0.61–1.45; 0.79)	1.16 (0.71–1.89; 0.56)	1.02 (0.57–1.82; 0.94)
**Baseline HIV RNA (per log_10_ copies/mL)**	**1.43 (1.26–1.62; <0.01)**	**1.32 (1.18–1.48; <0.01)**	**1.26 (1.10–1.45; <0.01)**	**1.36 (1.17–1.58; <0.01)**
**Baseline CD4+ T-cell count (per 100/mm^3^)**	**0.87 (0.81–0.93; <0.01)**	**0.90 (0.85–0.96; <0.01)**	**0.89 (0.82–0.95; <0.01)**	**0.88 (0.81–0.96; <0.01)**
**Baseline CD8+ T-cell count (per 100/mm^3^)**	0.98 (0.93–1.03; 0.35)	0.98 (0.93–1.02; 0.30)	0.97 (0.92–1.03; 0.32)	0.99 (0.94–1.06; 0.87)
**Baseline CD4/CD8 ratio**	**0.44 (0.26–0.7; <0.01)**	**0.62 (0.41–0.94; 0.02)**	**0.60 (0.37–0.97; 0.04)**	**0.45 (0.25–0.82; <0.01)**
**eGFR (ml/min)**				
60–90 mL/min	*Reference category*	*Reference category*	*Reference category*	*Reference category*
<60 mL/min	1.20 (0.54–2.68; 0.65)	**2.61 (1.45–4.70; <0.01)**	**4.06 (2.05–8.04; <0.01)**	1.43 (0.60–3.42; 0.42)
**FIB4 score**				
1.45–3.25	*Reference category*	*Reference category*	*Reference category*	*Reference category*
>3.25	**6.96 (3.06–15.82; <0.01)**	**5.27 (2.58–10.78; <0.01)**	**6.50 (2.87–14.72; <0.01)**	**6.05 (2.40–15.24; <0.01)**
**Baseline Framingham score (per unit)**	0.94 (0.89–1.00; 0.07)	0.96 (0.91–1.03; 0.24)	**0.93 (0.87–0.99; 0.03)**	**0.92 (0.86–0.98; 0.02)**
**Baseline cancer diagnosis**				
NO	*Reference category*	*Reference category*	*Reference category*	*Reference category*
YES	1.55 (0.80–3.00; 0.20)	1.35 (0.74–2.46; 0.33)	1.64 (0.84–3.19; 0.15)	**2.29 (1.16–4.54; 0.02)**

cART, combination antiretroviral treatment; NRTI, nucleos(t)ide reverse transcriptase inhibitors; NNRTI, non-nucleos(t)ide reverse transcriptase inhibitors; INSTI, integrase strand inhibitors; PI, protease inhibitors; RPV, rilpivirine; TXF, tenofovir alafenamide or tenofovir disoproxil fumarate; XTC, emtricitabine or lamivudine; 3TC, lamivudine; ABC, abacavir; DRV, darunavir; COBI, cobicistat; BOOST, ritonavir-boosted regimens; PWID, people who inject drugs; MSM, men who have sex with men; eGFR, estimated Glomerular Filtration Rate; HBsAg, hepatitis B antigen; HCVAb, hepatitis C antibodies. Statistically significant values are displayed in bold.

**Table 3:** Hazards of treatment interruption in relation to each of the outcome events (multivariable analysis).

	Hazards of Interruption (95% Confidence Interval; *p*-Value) Due to:			
	Efficacy	Convenience	Safety	Durability
**cART backbone**				
3TC ^a^	*Reference category*	*Reference category*	*Reference category*	*Reference category*
ABC/XTC	1.37 (0.66–2.85; 0.40)	1.19 (0.65–2.16; 0.57)	0.95 (0.44–2.05; 0.90)	1.47 (0.61–3.58; 0.39)
DRV-boosted	0.62 (0.13–2.98; 0.55)	0.78 (0.27–2.26; 0.64)	0.64 (0.17–2.46; 0.52)	0.45 (0.05–3.90; 0.46)
RPV	0.41 (0.11–1.54; 0.19)	0.71 (0.29–1.76; 0.46)	0.80 (0.27–2.36; 0.68)	0.19 (0.02–1.57; 0.12)
XTC/TXF	**4.25 (1.94–9.34; <0.01)**	**2.55 (1.30–5.00; <0.01)**	**3.61 (1.68–7.76; <0.01)**	**6.10 (2.42–15.38; <0.01)**
**Previous cART**				
Naïve ^b^	*Reference category*	*Reference category*	*Reference category*	*Reference category*
NRTI/INSTI	0.52 (0.17–1.54; 0.23)	0.69 (0.29–1.64; 0.40)	0.82 (0.32–2.13; 0.69)	0.45 (0.13–1.59; 0.22)
NRTI/NNRTI	**0.36 (0.18–0.70; <0.01)**	**0.37 (0.20–0.67; <0.01)**	**0.29 (0.14–0.62; <0.01)**	**0.34 (0.16–0.72; <0.01)**
NRTI/PI-boosted	**0.48 (0.26–0.87; 0.01)**	**0.50 (0.30–0.84; <0.01)**	**0.48 (0.26–0.87; 0.02)**	**0.40 (0.20–0.79; <0.01)**
Other	0.55 (0.28–1.08; 0.08)	0.63 (0.35–1.14; 0.13)	0.61 (0.31–1.21; 0.16)	**0.42 (0.19–0.94; 0.03)**
**Baseline HIV RNA (per log_10_ copies/mL) ^c^**	**1.35 (1.14–1.60; <0.01)**	**1.28 (1.10–1.50; <0.01)**	1.15 (0.95–1.38; 0.15)	**1.31 (1.07–1.60; <0.01)**
**Baseline CD4** **+ T-cell count (per 100/mm^3^) ^d^**	0.95 (0.88–1.03; 0.23)	0.96 (0.90–1.03; 0.30)	0.92 (0.84–1.00; 0.06)	0.95 (0.87–1.05; 0.32)
**Baseline CD8** **+ T-cell count (per 100/mm^3^) ^e^**	0.99 (0.94–1.04; 0.67)	0.99 (0.95–1.03; 0.60)	1.00 (0.95–1.05; 0.85)	1.01 (0.95–1.07; 0.76)

Adjustments for confounders were determined by the generalized adjustment criterion on an expert-based causal graph: ^a^ CD4+ T-cell count, FIB4 score, Framingham score, HBsAg, HCV Ab, HIV RNA, age, antihypertensive drugs, antidepressants drugs, cancer, eGFR, blood glucose, prior cART, risk group, statin use, blood triglycerides; ^b^ age; ^c^ CD4+ T-cell count, age; ^d^ HIV RNA, age; ^e^ CD4+ T-cell count, HIV RNA. cART, combination antiretroviral treatment; NRTI, nucleos(t)ide reverse transcriptase inhibitors; NNRTI, non-nucleos(t)ide reverse transcriptase inhibitors; INSTI, integrase strand inhibitors; PI, protease inhibitors; RPV, rilpivirine; TXF, tenofovir alafenamide or tenofovir disoproxil fumarate; XTC, emtricitabine or lamivudine; 3TC, lamivudine; ABC, abacavir; DRV, darunavir; COBI, cobicistat; BOOST, ritonavir-boosted regimens. Statistically significant values are displayed in bold.

**Table 4:** Summary of the risk and protective factors associated with the study metrics.

	Efficacy	Convenience	Safety	Durability
**Tenofovir-containing regimens**	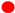	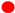	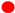	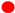
**Naïve status**	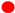	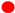	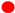	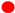
**Prior cART not containing an INSTI**	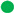	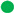	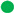	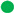
**HBsAg positive carriers**	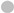	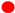	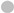	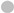
**Baseline HIV RNA**	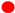	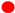	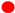	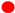
**Baseline CD4** **+ T-cell count**	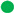	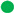	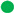	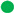
**Baseline CD4/CD8 ratio**	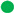	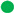	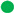	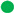
**eGFR < 60 mL/min**	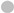	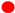	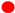	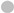
**FIB4 score > 3.25**	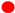	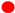	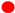	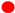
**Baseline Framingham score**	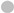	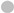	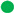	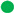
**Baseline cancer diagnosis**	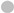	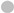	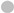	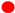

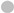
 Not a statistically significant association; 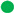 protective factor; 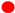 risk factor; cART, combination antiretroviral treatment; INSTI, integrase strand inhibitors; HBsAg, hepatitis B antigen; eGFR, estimated Glomerular Filtration Rate.

## Data Availability

The data can be made available by submitting a request to the Malattie Infettive e Salute Internazionale Foundation (MISI, translation: Infectious Diseases and International Health Foundation) in Brescia, Italy (presidenza@fondazionemisi.it).

## Supplementary Materials

The following supporting information can be downloaded at: https://www.mdpi.com/article/10.3390/v15040924/s1, Figure S1: Directed acyclic graph representing causal dependencies among study variables, exposures and outcome(s); Table S1: Characteristics of other previous cART regimens performed by experienced participants; Table S2: Characteristics of experienced participants; Table S3: Characteristics of naïve participants.
