# Supplementary material for: Efficacy, Convenience, Safety and Durability of DTG-Based Antiretroviral Therapies: Evidence from a Prospective Study by the Italian MaSTER Cohort
Source: Viruses. 2023 Apr 6;15(4):924. doi: 10.3390/v15040924 (PMC10145592; doi:10.3390/v15040924)
Supplement: Supplementary file 1 [file viruses-15-00924-s001.zip › viruses-2223371-supplementary.pdf]

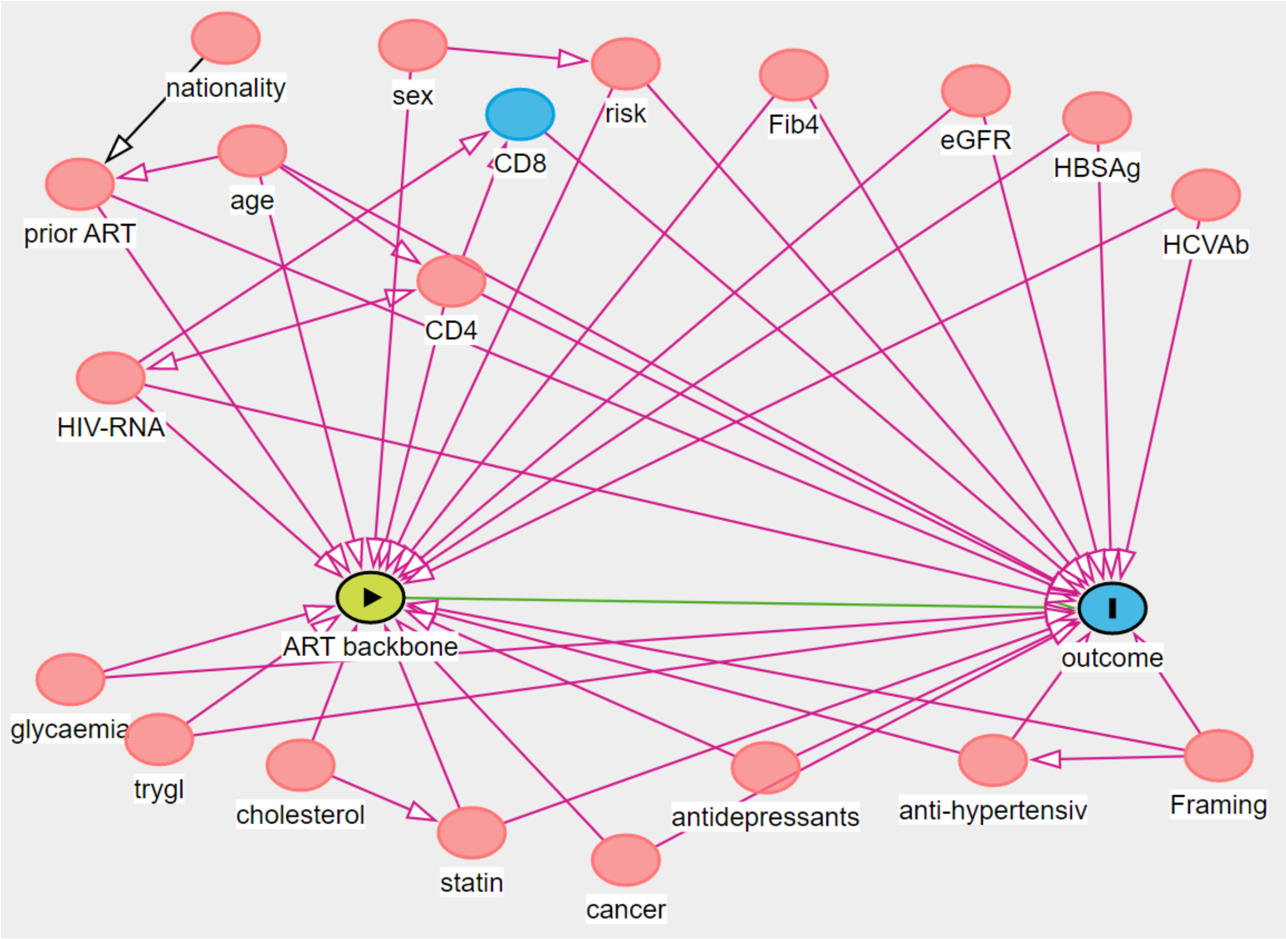

Figure S1. Directed acyclic graph representing causal dependencies among study variables, exposures and outcome(s).

Table S1. Characteristics of other previous cART regimens performed by experienced participants.

Other prior cART (N=68)	N°	%
PI-boosted	14	20.6%
NRTI/INSTI/PI-boosted	13	19.1%
NRTI/NNRTI/INSTI/PI-boosted	10	14.7%
NRTI/NNRTI/PI-boosted	8	11.8%
INSTI/PI-boosted	7	10.3%
NRTI	5	7.3%
NNRTI/PI-boosted	3	4.4%
Other	8	11.8%

Table S2. Characteristics of experienced participants.

Characteristics of experienced participants (N=300)	N° or Median	% or (IQR)
**Age (years)**	54	(47-58)
**Female sex**	77	25.7%
**Country (Italy)**	255	85.0%
**Year of starting DTG**		
2018	19	6.3%
2019	239	79.7%
2020	39	13.0%
2021	3	1.0%
**cART backbone**		
XTC/TXF	38	12.7%
3TC	96	32.0%
XTC/ABC	113	37.7%
RPV	35	11.7%
DRV/RTV	18	6.0%
**Mode of transmission**		
Other	21	7.0%
MSM	79	26.3%
IDU	78	26.0%
Heterosexual	122	40.7%
**HBsAg chronic carriers**	7	2.3%
**Positive HCV Ab participants**	89	29.7%
**Prior/baseline cancer**	24	8.0%
**Baseline HIV RNA Log_{10} copies/mL**	1.7	(1.7-1.7)
**Baseline CD4 T-cells/mm^3**	700	(536-897)
**Baseline CD8 T-cells/mm^3**	808	(589-1114)
**Baseline CD4/CD8 ratio**	0.83	(0.59-1.18)
**Baseline FIB-4 score**	1.08	(0.76-1.55)
**Baseline eGFR (ml/min)**	90.38	(76.27-101.87)
**Baseline Framingham score**	13	(10-15)
**Death**	1	0.3%
**Total follow up time (days)**	597.5	(360.75-744)

Table S3. Characteristics of naïve participants.

Characteristics of naïve participants (N=71)	N° or Median	% or (IQR)
**Age (years)**	48	(39-55)
**Female sex**	15	21.1%
**Country (Italy)**	54	76.1%
**Year of starting DTG**		
2018	2	2.8%
2019	58	81.7%
2020	3	4.2%
2021	8	11.3%
**cART backbone**		
XTC/TXF	42	59.2%
3TC	10	14.1%
XTC/ABC	16	22.5%
RPV	1	1.4%
DRV/RTV	2	2.8%
**Mode of transmission**		
Other	4	5.6%
MSM	23	32.4%
IDU	2	2.8%
Heterosexual	42	59.2%
**HBsAg chronic carriers**	0	0.0%
**Positive HCV Ab participants**	6	8.5%
**Prior/baseline cancer**	9	12.7%
**Baseline HIV RNA Log_{10} copies/mL**	5.05	(4.4-5.38)
**Baseline CD4 T-cells/mm^3**	207	(54-463)
**Baseline CD8 T-cells/mm^3**	805	(486-1092)
**Baseline CD4/CD8 ratio**	0.18	(0.09-0.52)
**Baseline FIB-4 score**	1.01	(0.62-1.54)
**Baseline eGFR (ml/min)**	100.3	(86.38-109.41)
**Baseline Framingham score**	9	(4-12)
**Death**	0	0.0%
**Total follow up time (days)**	363	(252-645)
